# Supplementary material for: SCOPE-Seq: a scalable technology for linking live cell imaging and single-cell RNA sequencing
Source: Genome Biol. 2018 Dec 24;19:227. doi: 10.1186/s13059-018-1607-x (PMC6305572; doi:10.1186/s13059-018-1607-x)
Supplement: Supplementary file 1 — Supplementary methods and figures. (DOCX 2535 kb) [file 13059_2018_1607_MOESM1_ESM.docx]

1. **Supplementary Methods**

*Production of dual-barcoded mRNA capture beads*

**Fig. 1A** shows the bead production workflow. Approximately 700,000 commercially available “Drop-Seq” beads (Macosko-2011-10(V+), ChemGenes) are washed and re-suspended in 1280 uL of nuclease-free water and then immediately evenly distributed into 64 wells of a 96-well plate. The plate is then sealed with an adhesive cover, vortexed for 1 minute, sonicated in a water bath sonicator (FS-20, Fisher Scientific) for 5 minutes, and incubated at 4°C for 12 hours. The supernatant from each well (containing DNA that has detached from the beads, which we refer to as bead-free DNA) is transferred into a separate plate and stored in a -20°C freezer. Beads in each well are then washed again with nuclease-free water. A unique combination of oligonucleotides (4 µM each) selected from a set of 6 oligonucleotides which we refer to as optical barcode oligonucleotides (OBOs) and a ligation reaction mixture (1x T4 RNA ligase reaction buffer, 1 mM ATP, 25% PEG 8000, 0.1% Tween-20, 0.7 U/µL T4 RNA ligase 1 (M0204S, New England BioLabs)) are sequentially added to each of the 64 wells. The plate is then incubated at room temperature for 24 hours on a rotisserie. 5 µL of 10% sodium dodecyl sulfate (SDS) and 1 µL of 500 mM EDTA are then added to each well, mixed and incubated for 5 minutes. The beads from all 64 wells are then pooled into a single tube, washed in TE/TW buffer (10 mM Tris pH 8.0, 1 mM EDTA, 0.01% Tween-20) and subjected to a second round of the above reactions with another set of 6 oligonucleotide species which produces another plate of bead-free DNA-containing supernatant. At the end of this process, each bead will have one of 2^6^ x 2^6^ = 4,096 possible combinations of the 12 OBOs attached to it. We refer to these OBO combinations as optical barcodes. The two sets of bead-free DNA, which contain the cell-identifying sequence barcode associated with each bead in each well, allows us to track which beads become associated with each of these 4,096 optical barcodes.

*Bead-free DNA library construction, sequencing, and analysis*

5 µL of each bead-free DNA containing supernatant is mixed with 10 µL 1 µM NexterapolyA oligonucleotide and 2 µL 10X NEBuffer 2 (B7002S, New England BioLabs). The mixtures are incubated at 95°C for 2 minutes and then cooled to room temperature. 2 µL 10 mM dNTPs and 1 µL Klenow Fragment (M0212S, New England BioLabs) are then added to each mixture and incubated at room temperature for 15 minutes, and at 37°C for 1 hour, followed by heat inactivation at 75°C for 20 minutes, and then stored at -20°C. 4µL from each reaction is then further mixed with 2 µL 2 µM supernatant-specific P7 primer, 2 µL 2 uµM universal P5 primer, and 8 µL of PCR ready mix (KK2601, Kapa Biosystems). The reaction mixtures are then subjected to PCR amplification (95°C 3 minutes, 14 cycles of (95°C 10 seconds, 55°C 30 seconds, 72°C 30 seconds), 72°C 5 minutes). 5 µL of PCR product from each reaction is then pooled and purified first using the solid-phase reversible immobilization (SPRI) paramagnetic bead technology (A63880, Beckman Coulter) with a 2:1 bead-to-sample volume ratio, then using gel electrophoresis with a 10% TBE polyacrylamide gel (4565014, Bio-Rad). 1X SYBR Gold nucleic acid gel stain (S11494, ThermoFisher Scientific) is used to stain the DNA bands and the ~ 180bp bands (**Additional file 1 : Fig. S2A**) were extracted. The gel purified libraries are further characterized on an Agilent Bioanalyzer (**Additional file 1 : Fig. S2B**). The purified bead-free libraries are spiked with 40% PhiX control library (FC-110-3001, Illumina) and sequenced on a NextSeq 500 sequencer (Illumina) with a NextSeq 500/550 High Output v2 kit (75 cycle, FC-404-2005, Illumina) per the vendor’s instructions. A 26-base read 1 and an 8-base index 1 reads are obtained. A custom read 1 sequencing primer is used (**Additional file 4: Table S2)**. The 12-nt cell-identifying barcode and 8-nt index sequences are extracted from each of the resulting read. For each cell barcode that has an associated RNA-Seq profile, its abundance in each of the 128 bead-free libraries is counted. Error correction is performed on the index sequences when they are within a Hamming distance of one from any of the bead-free library index sequences.

*Single-cell RNA-Seq library construction, sequencing, and analysis*

U87 human and 3T3 mouse cells are cultured separately in Dulbecco’s modified eagle medium (DMEM, 11965118, Life Technologies) supplemented with 10% fetal bovine serum (FBS, 16000044, Life Technologies). On the day before experiment, a microwell array device is filled with wash buffer (20 mM Tris-HCl pH7.9, 50 mM NaCl, 0.1% Tween-20) and stored in a humid chamber (an empty pipette tip box filled with water). On the day of the experiment, U87 human and 3T3 mouse cells are separately stained respectively with green (C3100MP, ThermoFisher Scientific) and orange (C34851, ThermoFisher Scientific) live stain dyes in culture medium at 37^o^C for 15 minutes. The stained cells are then dissociated into single cell suspension and re-suspended in TBS buffer. The pre-filled microwell array device is flushed with TBS buffer. The dissociated human and mouse cells with a total cell concentration of 1700 cells/µL are then pipetted into the microwell array device. The cells are allowed to settle into the microwells for 3 minutes. Any un-trapped cells are then flushed out with TBS buffer. The cell-loaded microwell array device is then scanned under a microscope in two fluorescence channels that corresponds to the two live stains used and the bright field channel. Dual-barcoded mRNA capture beads (500 beads/µL) are then loaded to the microwells followed by a TBS buffer flush. The cell and bead-loaded device is then connected to a computer-controlled reagent delivery and temperature control system. Lysis buffer (1% 2-Mercaptoethanol (BP176-100, Fisher Scientific), 99% Buffer TCL (1031576, Qiagen)) and perfluorinated oil (F3556-25ML, Sigma-Aldrich) are infused through the device in continuously and in rapid succession to physically isolate individual microwells and lyse the trapped cells. The device is kept at 50^o^C for 20 minutes to further promote cell lysis, and then at 25^o^C for 90 minutes for mRNA capture. To determine whether cells are lost or moving in the microwell array during this process, we compared fluorescence images of the cells in the microwells taken after cell loading and after cell lysis (**Additional file 1 : Fig. S3**). We find that most (~99.8%) cells stayed in their original microwells and that ~0.2% of cells were lost. Wash buffer supplemented with RNase inhibitor (0.02 U/µL SUPERaseIN (AM2696, Thermo Fisher Scientific) in wash buffer) is then flushed through the device to unseal the microwells and remove any uncaptured mRNA molecules. Reverse transcription mixture (1X Maxima RT buffer, 1 mM dNTPs, 1 U/µL SUPERaseIN, 2.5 µM template switch oligo, 10 U/µL Maxima H Minus reverse transcriptase (EP0752, Thermo Fisher Scientific), 0.1% Tween-20) is infused into the device followed by incubation at 25^o^C for 30 minutes and then at 42^o^C for 90 minutes. A mixture of 12 unlabeled oligonucleotides whose sequences are complementary to those of the OBOs (1 µM each in wash buffer) are infused into the microwell array device, incubated for 1 hour at room temperature, followed by wash buffer. Exonuclease I reaction mixture (1X Exo-I buffer, 1 U/µL Exo-I (M0293L, New England Biolabs)) is then infused into the device and incubated at 37^o^C for 45 minutes, followed by sequential rinses with TE/TW buffer (10 mM Tris-HCl, 1 mM EDTA, 0.01% Tween-20, pH 8.0) and 150 mM NaOH solution. The device is then subjected to the optical demultiplexing workflow described in the next section. After optical demultiplexing, the device is cut into 10 separate regions. To facilitate device cutting, a new chip design (**Additional file 2 and 3**) is used. Microwells are arranged into 10 separated regions with sufficient space between them to accommodate cutting by a razor blade. Beads from each region are extracted by soaking each small piece of bead-containing PDMS in 100% ethanol, followed by vortexing, water bath sonication, and centrifugation in a 1.7 mL microcentrifuge tube. The beads are pelleted to the bottom of the tube while the PDMS piece is stuck at a different level of the tube thus achieving separation between the bead pellet and the PDMS piece (**Additional file 1 : Fig. S4**). The PDMS slab is then removed with a tweezer. Beads extracted from each region are processed in separate reactions for the downstream library construction steps. The extracted beads are then washed sequentially with 150 mM NaOH, TE/SDS buffer (10 mM Tris-HCl, 1 mM EDTA, 0.5% SDS), TE/TW buffer, and nuclease-free water and split into two 50 µL PCR reactions (1X Hifi Hot Start Ready mix (KK2601, Kapa Biosystems), 1 µM SMRTpcr primer (**Additional file 4 : Table S2**)) per region. 12 amplification cycles (95^o^C 3 min, 4 cycles of (98^o^C 20s, 65^o^C 45 s, 72^o^C 3 min), 8 cycles of (98^o^C 20s, 67^o^C 20s, 72^o^C 3min), 72^o^C 5 min) are performed on a thermocycler. PCR product from each region is pooled and purified using SPRI paramagnetic bead technology with a bead-to-sample volume ratio of 0.6:1. Purified cDNA are then tagmented and further amplified using the Nextera kit for *in vitro* transposition (FC-131-1024, Illumina). 0.6 ng cDNA is used as input per reaction. A unique i7 index primer is used to barcode the libraries obtained from each region of the device. The i5 index primer is replaced by a universal P5 primer (**Additional file 4 : Table S2)** for the selective amplification of 5’ end of cDNA (corresponding to the 3’ end of mRNA). Two rounds of SPRI paramagnetic bead-based purification with a bead-to-sample volume ratio of 0.6:1 and 1:1 respectively are performed sequentially on the Nextera PCR product to obtain sequencing-ready libraries. The resulting single-cell RNA-Seq libraries are pooled, spiked with 20% PhiX library (FC-110-3001, Illumina), and sequenced on a sequencer (NextSeq 500, Illumina) with a 21-cycle read 1, 66 cycle read 2, and an 8 cycle index read. A custom sequencing primer (**Additional file 4 : Table S2)** is used for read 1.

The resulting sequencing reads are then demultiplexed, aligned, and quantified as described previously[^16^](#_ENREF_16). Briefly, we first aligned the reads to a merged human/mouse genome (GRCh38 for human and GRCm38 for mouse) with merged GENCODE transcriptome annotations (GENCODE v.24 for both species) using STAR v.2.5.0 after removing 3’ poly(A) tails (indicated by tracts of >7 A’s) and fragments with fewer than 24 nucleotides after poly(A) tail removal. We then assigned an address to each read with a unique, strand-specific alignment to exonic sequence comprised of the cell-identifying barcode, unique molecular identifier (UMI) barcode, and gene identifier. Finally, we collapsed amplification duplicates using the UMIs and corrected errors in both the cell-identifying and UMI barcodes to generate a preliminary matrix of molecular counts for each cell as described previously^16^. We filtered the cell-identifying barcodes to avoid dead cells and other artifacts as described in Yuan *el al*^16^. We removed all cell-identifying barcodes for which >10% of molecules aligned to genes expressed from the mitochondrial genome or for which the ratio of molecules aligning to whole gene bodies (including introns) to molecules aligning exclusively to exons was >1.5. Finally, we also removed cell-identifying barcodes for which the number of reads per molecule or number of molecules per gene deviated by >2.5 standard deviations above the mean.

*Optical demultiplexing workflow, system design, and image processing*

Twelve rounds of sequential fluorescence hybridization (SFH) are used to identify the set of OBOs attached to each bead. Each round of SFH consists of four major steps including a background scan, probe hybridization, probe scan, and probe stripping. During both background and probe scans, the bead-containing microwell array is imaged in wash buffer through both the fluorescence and bright field channels. During probe hybridization and probe stripping, a hybridization mixture (probe oligonucleotides at 200 nM in wash buffer) and a melting solution (150 mM NaOH) are flowed into the device, respectively, and incubated at room temperature for 10 minutes followed by rinsing with wash buffer. Probes used in each hybridization mixture consist of Cy5-labeled oligonucleotides whose sequences are complementary to each of the 12 OBOs (**Additional file 4 : Table S2**). An automated reagent delivery and scanning system (**Additional file 1 : Fig. S5**) is designed for automated optical demultiplexing. In the automated reagent delivery system, fixed positive pressure (~1 psi) stabilized by a pressure regular (AW20-F02, SMC Pneumatics) is used to drive fluid flow. Two 10-channel rotary selector valves (MLP778-605, IDEX Health & Science) are connected in parallel to toggle between 14 reagent channels. A three-way solenoid valve (EW-01540-11, Cole-Parmer), located downstream of the microwell array device, is used as an on/off switch for reagent flow. The microwell array device is constantly pressurized during incubation steps, which helps prevent evaporation and suppresses bubble formation. A microfluidic flow cell device is connected in-series immediately upstream of the microwell array device. The flow cell device traps bubbles, preventing them from entering the microwell array device. A water hammer arrestor consisting of a half-filled cryotube is connected immediately downstream of the microwell array device and upstream of the on-off flow switch valve to buffer the hydraulic shock produced by actuation of the on-off switch valve. The 10-channel selector valves are controlled by a USB digital I/O device (NI USB-6501, National Instruments). The three-way solenoid valve is controlled by the same USB digital I/O device, but through a homemade transistor-switch circuit.

The automated scanning system consists of an inverted epifluorescence microscope (Eclipse Ti-U, Nikon) equipped with a motorized stage and filter wheel, and an EM-CCD camera (iXon3, Andor). A C program is used to control both the reagent delivery system and the scanning system. A glass slide is attached to the bottom surface of the microwell array device to keep the microwells in the specimen plane. The individual image tiles from each scan are stitched together. The stitched images from each scan are then registered to the first demultiplexing cycle scan. The outlines of individual microwells are then identified using the bright field images by first thresholding followed by object detection using the particle analyzer plugin in ImageJ. The average bright field and fluorescence intensities of each microwell in each scan are then measured. Two separate populations of microwells are observed based on their average bright field intensities in each demultiplexing cycle (**Additional file 1 : Fig. S6**). The population with higher average bright field intensity corresponds to empty microwells whereas the other population corresponds to microwells containing beads. The mean bright field intensity of each population is identified by fitting a bimodal Gaussian model to the average bright field intensity distribution. The intensity of the shortest bin between the two mean intensities is used as the threshold that separates the two microwell populations. The device is also scanned immediately after mRNA capture and after reverse transcription. The same analysis is performed on the bright field images to identify microwells with beads after cell lysis and reverse transcription (**Additional file 1 : Fig. S7**). Further analysis is then performed only on microwells with beads. The average fluorescence intensity of each microwell measured from the background scan is subtracted from that measured from the corresponding probe scan. The resulting background-subtracted fluorescence intensities exhibit a bimodal distribution as well (**Additional file 1 : Fig. S8, S9A**). The population with higher average fluorescence intensity corresponds to microwells with beads in the “on” state whereas the other population corresponds to microwells with beads in the “off” state. The thresholds that separate these two populations are identified using the same algorithm described above. As expected, the distribution of optical barcode usage closely follows Poisson statistics (**Additional file 1 : Fig. S9B**) suggesting a uniform representation of optical barcodes among the stock beads.

*Cell image processing/feature extraction*

Individual cell live stain image tiles from each color channel are stitched. Rolling ball background subtraction is performed on the stitched images. The background-subtracted images are then registered to the first demultiplexing cycle scan. The microwell outlines obtained in processing the demultipexing images are used to obtain the average live stain intensities of cells in each microwell in each of the two live stain color channels. Two separate populations of microwells are observed based on cell live stain intensities (**Additional file 1 : Fig. S10**). The population with higher intensity corresponds to microwells with cells stained in the corresponding color whereas the other population corresponds to microwells without cells stained in the corresponding color. The thresholds that separate these two populations are identified using the same algorithm used in the processing of demultiplexing images. These microwell-averaged cell live stain intensities are used to decide the species identity of the cell in each microwell. Further analysis is then performed only on microwells with either U87 human cells or 3T3 mouse cells. To obtain imaging features of individual cells, adaptive thresholding is performed on the fluorescence live stain images. The adaptive threshold for each microwell is identified based only on the intensity distribution of the pixels within the smallest bounding square of that microwell. The default (auto) threshold identification algorithm in ImageJ is used. The outline of each cell is then identified using the particle analyzer in ImageJ. Microwells with more than one cell detected are excluded from analysis. 19 imaging features are then obtained for each cell using their live stain image. These features are area, mean intensity, standard deviation of intensity, mode of intensity, minimum intensity, maximum intensity, perimeter, major axis, minor axis, circularity, Feret’s diameter, integrated intensity, median intensity, skewness, kurtosis, minimum Feret’s diameter, aspect ratio, roundness, and solidity.

*Linking imaging and sequencing data*

An accurate optical barcode-to-sequencing barcode look-up table is also critical. We generate the look-up table by associating the unique combination of oligonucleotides added to each ligation reaction in each cycle of split-pool synthesis with the sequencing barcodes detected in the corresponding bead-free library. During each round of split-pool ligation, one of 2^6^ = 64 combinations of six oligonucleotides is added to each reaction, generating a 6-bit binary optical barcode for each split. Two rounds of split-pool ligation are performed thus yielding 12-bit binary optical barcodes. The sequencing barcode of each bead should be represented once among the 64 bead-free libraries from the first round of ligation and once among the second set. For each sequencing barcode in each round of split-pool synthesis, we ranked its normalized frequency in the 64 libraries in descending order and averaged the resulting vector across all sequencing barcodes (**Additional file 1 : Figure S9C-D**). As expected, the vast majority of sequencing barcodes are represented in only one of the 64 libraries for both rounds of ligation, suggesting only minor imperfections due to sequencing error and low levels of residual DNA between split-pool cycles. The signal-to-background ratios are 17.1 and 9.5 for the first and second rounds of split-pool reactions, respectively.

We used our mixed species experiment to assess the accuracy with which we could link sequencing and imaging data with SCOPE-seq. We first defined a set of singlets from the scRNA-Seq data as cells with >90% of reads aligning uniquely to the same species. Next, we extracted the sequencing barcodes for these singlets and identified the “bead-free” sequencing library indices that contained each cell barcode. There are two sets of “bead-free” sequencing library indices corresponding to the two cycles of split-pool synthesis used to generate the dual-barcoded beads. We assigned an optical barcode to each sequencing barcode by taking the index from each of the two sets with the most reads corresponding to the sequencing barcode. Taken together, the two indices identify a specific combination of OBOs comprising the optical barcode. We can link ~89.6% of sequencing barcodes to optical barcodes by this procedure. Finally, we determine whether the optical barcode is found in the optical demultiplexing data, and if it is, we determine the imaging-based species call for the cell in the corresponding microwell. We use the concordance between the scRNA-seq and imaging-based species calls to compute our accuracy.

*Imaging-based multiplet detection*

Two-color fluorescence images of microwells with cells from both species are merged into a monochrome image by summing the pixel intensities from the two-color channels (**Additional file 1 : Fig. S11**). The monochrome image is then examined by a researcher at multiple brightness levels. A multiplet is identified when there is more than one bright object separated by a clear dark boundary.

*Principal component and gene set enrichment analysis of the cell imaging features*

We used the singlet murine cell data set in our mixed species experiment to analyze the relationship between gene expression and the imaging features described above, because it contained more cells. We started with 805 murine cells and filtered this to 728 cells after removing cells in which the fluorescence containing saturated pixels. We then z-normalized the imaging feature matrix described above for these 728 cells and computed the principal components of the resulting matrix of z-scores. Next, we computed Pearson’s correlation coefficient between the principal component vector and the vector of gene expression levels for each gene in log(counts per million+1) for the first two principal components. We then ranked all of the protein-coding genes detected in at least 10% of cells by correlation with each of the two principal components and used this as input for gene set enrichment analysis (GSEA) using the gene sets from the Molecular Signatures Database C5 collection of cellular component gene ontologies (pre-ranked mode, “classic” enrichment statistic).

*Partial least square regression and gene set enrichment analysis of the cell imaging features*

We restricted our analysis on protein-coding genes that are detected in at least 10% of cells. We z-normalized the imaging feature matrix and the gene expression matrix. Partial least square regression (PLSR) was then performed on the z-normalized matrices using the *plsregress* function in MATLAB. This analysis generates a linear model (Y = XB) that specifies the relationship between cell imaging features (Y) and cell gene expression profiles (X). The matrix B contains the weights of all genes for all imaging features. We then ranked all genes by their weights for each imaging feature. This generated a ranked gene list for each imaging feature. We performed GSEA to interpret these ranked gene lists. The top 10 GO terms enriched at the top of each ranked gene list are summarized in **Additional file 4 : Table 1**.

1. **Supplementary Figures**


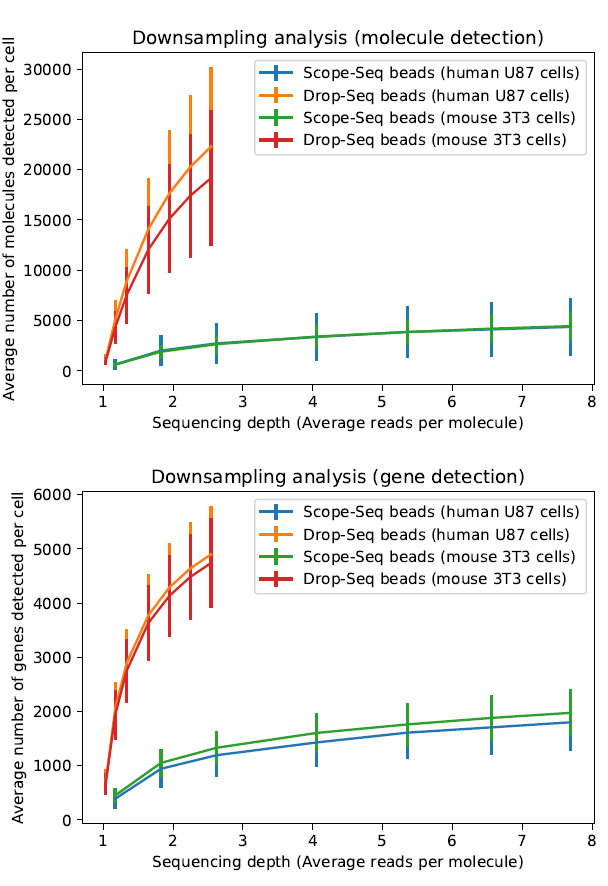


**Fig. S1.** Downsampling analysis of molecule (top) and gene (bottom) detection.


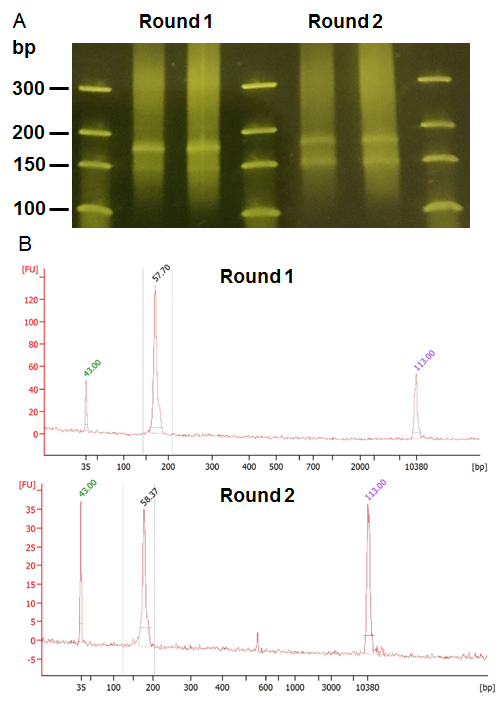


**Fig. S2.** (A) Gel electrophoresis of the bead-free libraries. (B) Bioanalyzer traces of the final purified bead-free libraries.


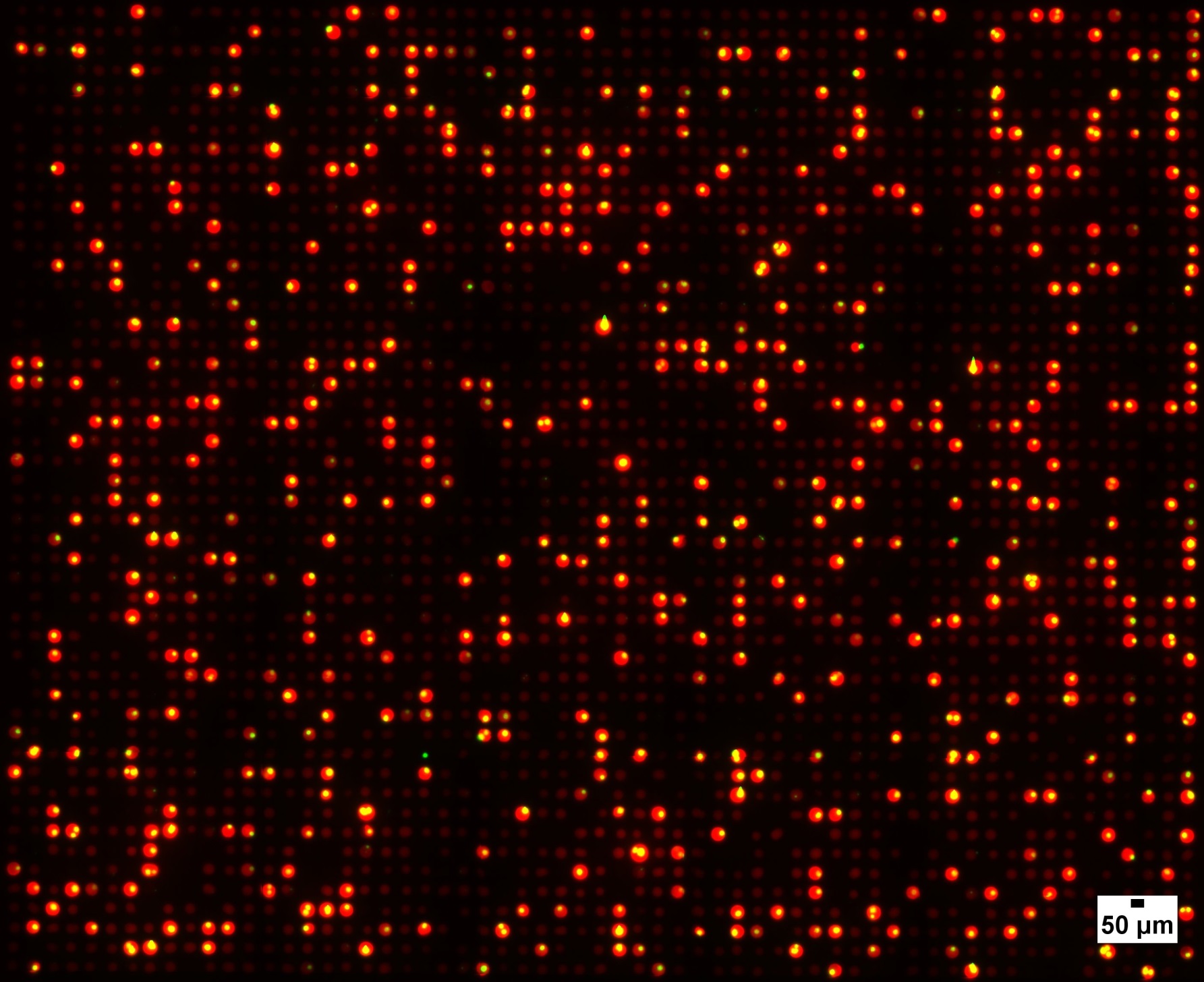


**Fig. S3.** Representative two-color composite image of cell occupancy in microwells. Before cell lysis (green channel), live stain fluorescence signal is restricted inside cells. After cell lysis (red channel), live stain fluorescence signal is released from the cells and fills up the whole microwells occupied by cells. Please note that the mRNA capture beads are slightly autofluorescence. This gives very dim signal in the red channel in microwells that don’t have a cell.


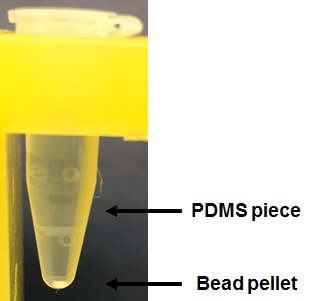


**Fig. S4.** Separation of PDMS piece and beads in a microcentrifuge tube.


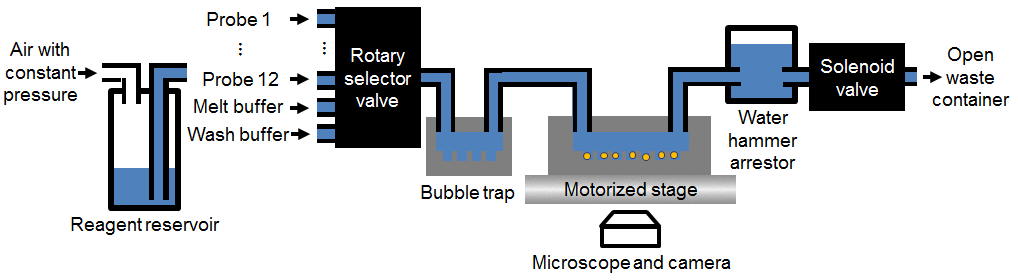


**Fig. S5.** Schematic of the reagent delivery system for automated optical demultiplexing.


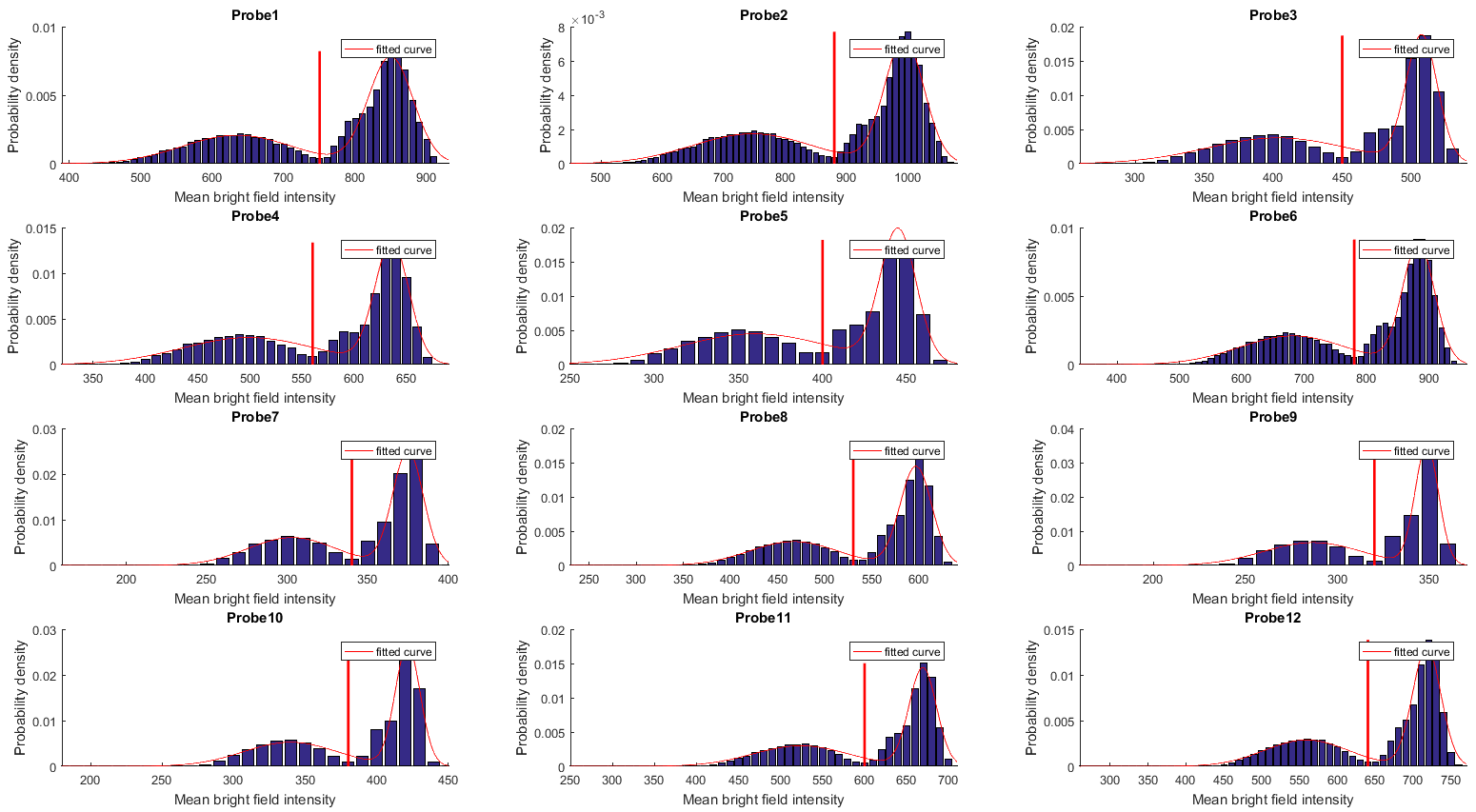


**Fig. S6.** Microwell mean bright field intensity distribution in each of the 12 demultiplexing cycles. Red curves are the fitted bimodal Gaussian distributions. Vertical red lines indicate the identified thresholds used to identify bead-containing microwells.


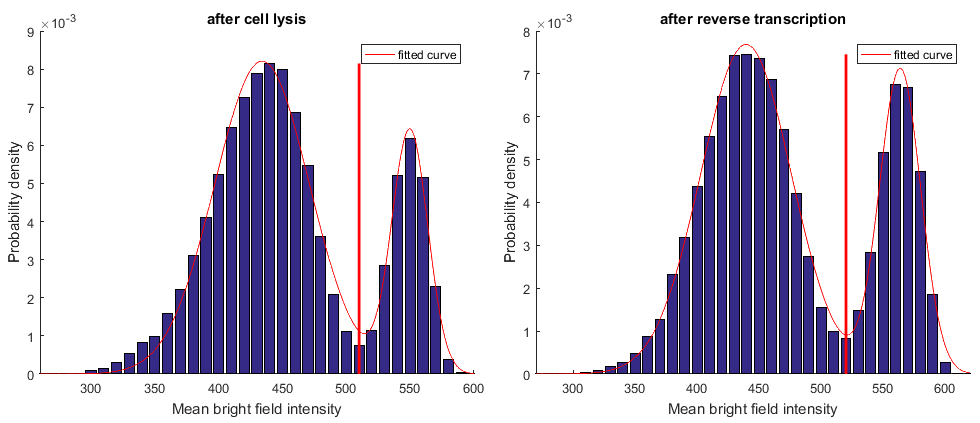


**Fig. S7.** Microwell mean bright field intensity distribution after cell lysis (left) and after reverse transcription (right). Red curves are the fitted bimodal Gaussian distributions. Vertical red lines indicate the identified thresholds.


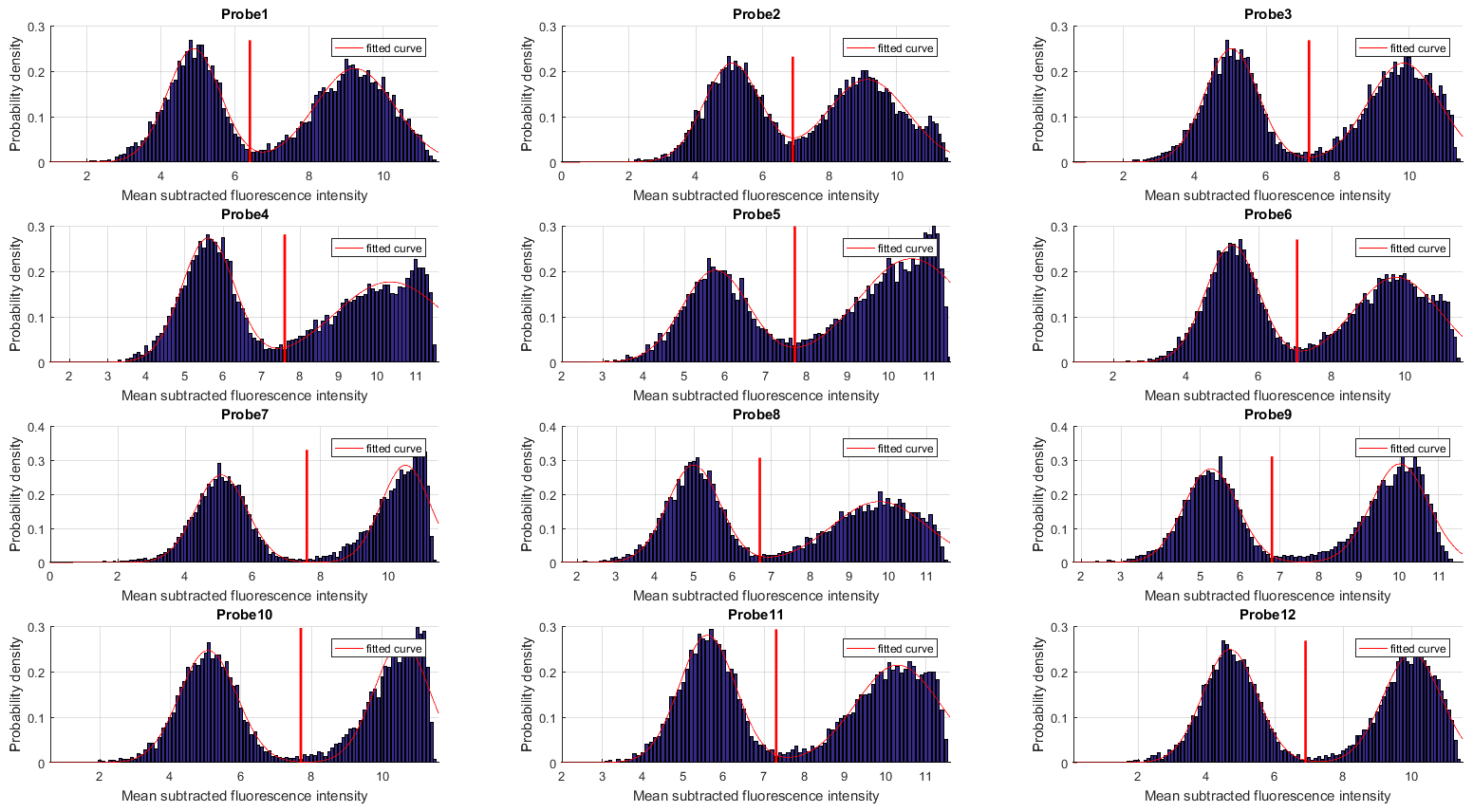


**Fig. S8.** Microwell mean-subtracted fluorescence intensity distribution in each of the 12 demultiplexing cycles. Red curves are the fitted bimodal Gaussian distributions. Vertical red lines indicate the identified thresholds.


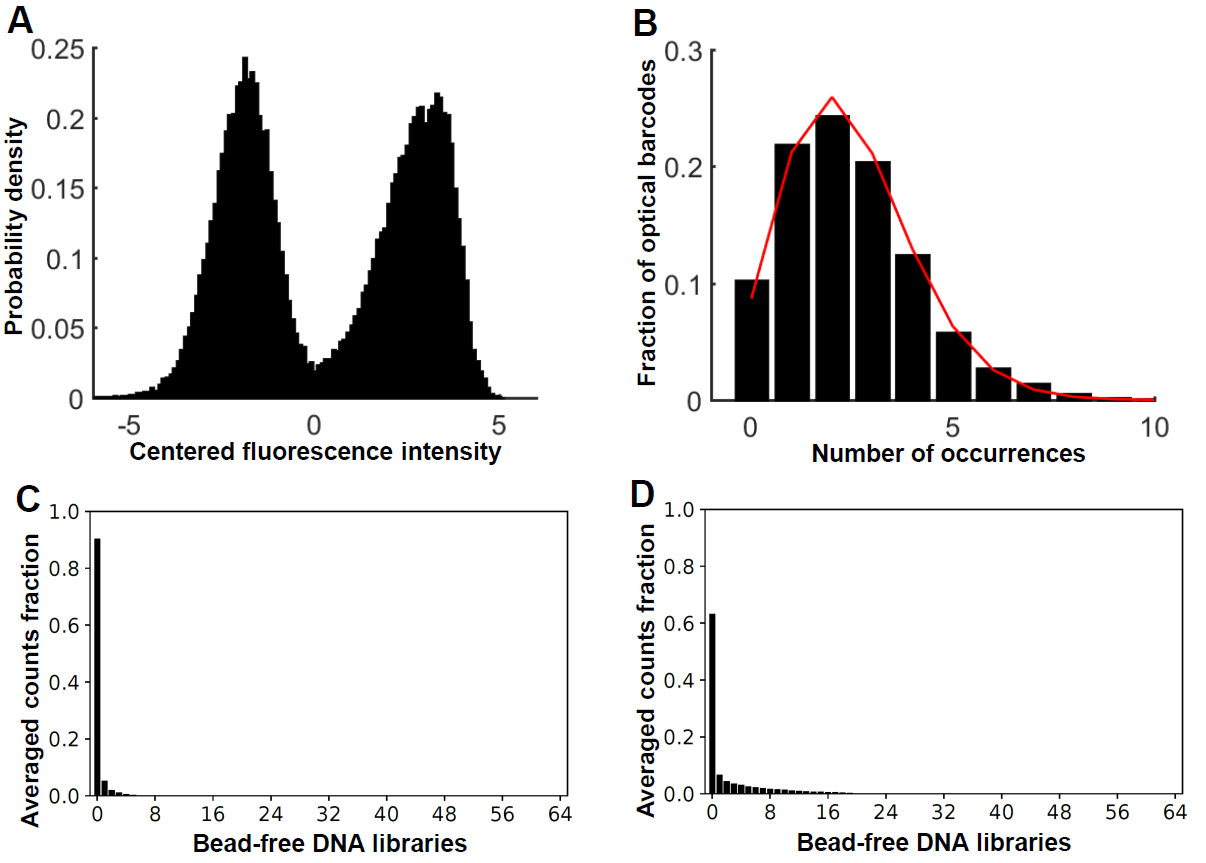


**Fig. S9.** A) Probability density of the centered bead fluorescence intensity during demultiplexing. B) Optical barcode usage histogram with fit to Poisson distribution (red). C) Averaged counts fraction distribution in the first round D) and second round of split-pool reactions.


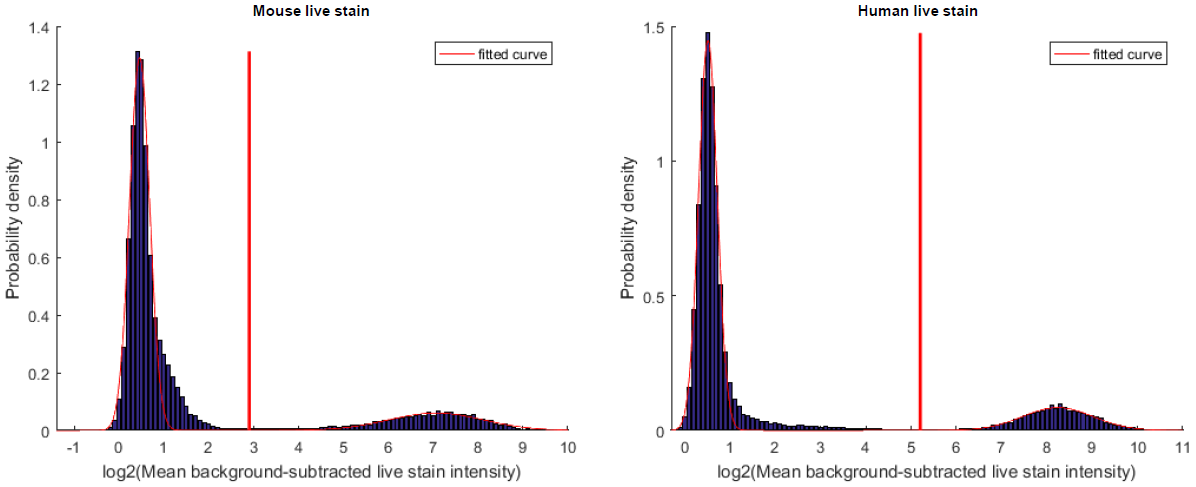


**Fig. S10.** Background-subtracted cell live stain intensity distributions. Red curves are the fitted bimodal Gaussian distributions. Vertical red lines indicate the identified thresholds.


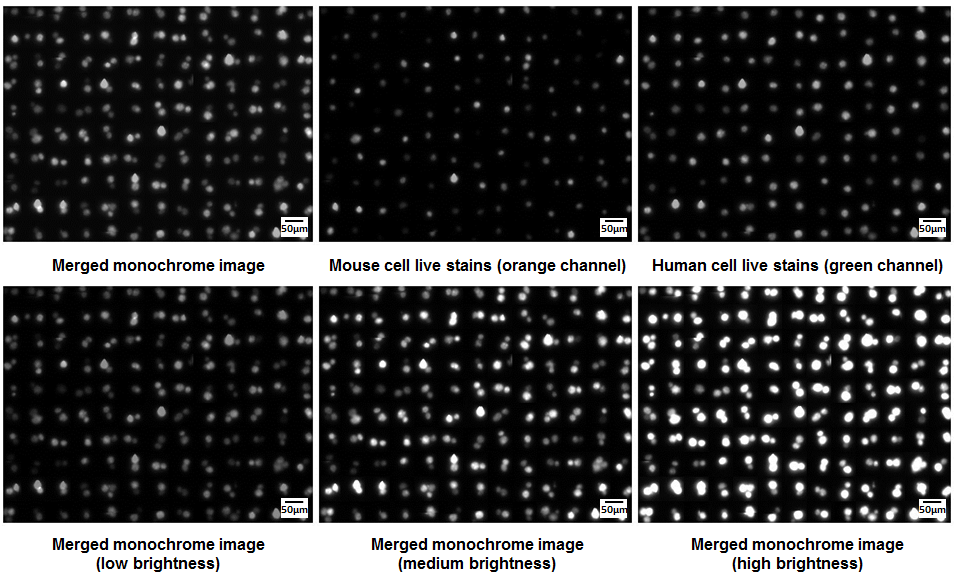


**Fig. S11.** Monochrome image merged from mouse cell live stain image and human cell live stain image. The monochrome image is viewed at multiple brightness level for multiplet detection.
